# Beyond tessellation: AI-derived choroidal vessel density on multi-channel cSLO as a surrogate biomarker for OCT choroidal parameters

**DOI:** 10.3389/fmed.2026.1884151

**Published:** 2026-10-07

**Authors:** Yuou Yao, Xi Wang, Tengfei Long, Chaohong Li, Saiguang Ling, Enzhong Jin, Yaoyao Sun, Shirong Su, Jianyu Xiong, Jiyang Tang, Xiang Meng, Yi Cai, Qiaozhu Zeng, Heng Li, Heng Miao

**Affiliations:** 1 Department of Ophthalmology, Peking University People’s Hospital, Beijing, China; 2 Beijing Key Laboratory of Ocular Disease and Optometry Science, Beijing, China; 3 EVision Technology (Beijing) Co. Ltd., Beijing, China; 4 Aerospace Information Research Institute, Chinese Academy of Sciences (CAS), Beijing, China; 5 Suzhou Microclear Medical Instruments Co., Ltd., Suzhou, Jiangsu, China; 6 Faculty of Biomedical Engineering, Shenzhen University of Advanced Technology, Shenzhen, China

**Keywords:** artificial intelligence, choroidal vessel, confocal scanning laser ophthalmoscopy, fundus image, optical coherence tomography

## Abstract

**Purpose:**

To develop an AI-based method for quantifying choroidal vessel density (CVD) from ultra-widefield confocal scanning laser ophthalmoscopy (UWF-cSLO) and to validate this metric against swept-source optical coherence tomography (SS-OCT) choroidal parameters.

**Methods:**

In this retrospective cross-sectional study, 633 eyes from 354 participants underwent simultaneous UWF-cSLO and SS-OCT imaging on an integrated platform, ensuring precise spatial co-registration. A TransUNet deep-learning model segmented choroidal vessels from red-channel (R; 665 nm) and infrared-channel (IR; 785 nm) images, yielding channel-specific metrics (R-CVD and IR-CVD) and a combined metric (IR + R CVD). Eight SS-OCT parameters—including choroidal thickness (CT), choroidal vascularity index (CVI), luminal area, stromal area (SA), total choroidal area (TCA), and vessel volume index (VVI)—served as reference measures. Associations were evaluated using Spearman correlations with participant-level clustered bootstrap confidence intervals, multivariable generalised estimating equations (GEE) adjusted for age and sex, and receiver operating characteristic (ROC) analysis.

**Results:**

Segmentation accuracy exceeded 0.95 (Dice coefficient) in both channels. R-CVD exhibited the strongest inverse correlations with structural and volumetric OCT parameters (SA: *ρ* = −0.619; CT: *ρ* = −0.615; TCA: *ρ* = −0.615; all *q* < 0.001), whereas IR + R CVD correlated most closely with proportion-based indices (VVI: *ρ* = −0.34; CVI: *ρ* = −0.29). These channel-dependent patterns persisted after adjustment for age and sex in multivariable GEE models. On ROC analysis, R-CVD discriminated thin from thick choroids with an area under the curve (AUC) of 0.789 (sensitivity 72.5%; specificity 74.8%), whereas IR-CVD alone showed near-chance performance (AUC 0.517).

**Conclusions:**

AI-derived CVD from UWF-cSLO correlates strongly with SS-OCT choroidal parameters in a wavelength-dependent manner and may serve as an accessible surrogate for choroidal structural assessment in settings without OCT.

## 1 Introduction

The choroid, the principal vascular layer of the posterior segment, supplies oxygen and nutrients to the outer retina and photoreceptors. Quantitative choroidal assessment has gained increasing relevance in myopia, pachychoroid spectrum disorders, and age-related macular degeneration. Swept-source optical coherence tomography (SS-OCT) affords deep tissue penetration and high axial resolution, making it the current reference standard for measuring choroidal parameters such as choroidal thickness (CT), luminal area (LA), stromal area (SA), total choroidal area (TCA), and the choroidal vascularity index (CVI) (1, 2). Defined as the ratio of luminal to total choroidal area, CVI has proved more stable and reproducible than CT alone (2–5). SS-OCT, however, remains costly and largely confined to tertiary referral centers, limiting population-level choroidal screening. Conventional color fundus photography (CFP) offers a more accessible alternative, yet it captures only the “shadow” of choroidal vessels transmitted through the retinal pigment epithelium (RPE)—a pattern termed fundus tessellation. Tessellation-derived indices, including pixel-based RGB ratios and AI-derived fundus tessellated density (FTD), correlate with subfoveal CT, CVI, LA, and TCA, all of which decline with increasing tessellation grade (6–9). CFP nonetheless provides limited depth selectivity: tessellation metrics reflect a composite trans-retinal signal rather than a targeted assessment of the choroidal vasculature.

Confocal scanning laser ophthalmoscopy (cSLO) addresses several of these limitations. The technique is widely available, non-contact, and capable of ultra-widefield (UWF) acquisition spanning up to 135°. Its multi-channel design employs discrete laser wavelengths to probe fundus structures at graded tissue depths (10, 11). The red channel (R; 665 nm) and infrared channel (IR; 785 nm) use longer wavelengths that penetrate more deeply than the broadband white-light sources of conventional fundus cameras and are less attenuated by RPE melanin absorption and light scattering (12). Consequently, these channels can image beyond the RPE into the choroidal stroma and large-vessel layer, revealing vascular features that remain obscured on standard CFP and tessellation-based metrics. Despite these optical advantages, existing cSLO studies of the choroid have relied on qualitative grading or single-metric semi-quantitative approaches; no validated method for automated, quantitative choroidal vessel assessment from cSLO images has been reported.

We therefore developed a TransUNet-based segmentation model to identify choroidal vessels automatically in R-channel and IR-channel UWF-cSLO images and to derive a quantitative metric, choroidal vessel density (CVD). Channel-specific CVD values (R-CVD and IR-CVD) were computed independently, and a combined metric (IR + R CVD) was obtained by summing the two values. We then examined correlations between cSLO-derived CVD and choroidal structural parameters measured by SS-OCT, with the aim of determining whether these readily obtainable cSLO metrics can serve as accessible indicators of choroidal structural status—potentially extending meaningful choroidal evaluation beyond specialist OCT-equipped centers.

## 2 Methods

### 2.1 Study design and ethical approval

This retrospective, cross-sectional observational study was conducted in accordance with the tenets of the Declaration of Helsinki. The protocol was approved by the Institutional Review Board of Peking University People's Hospital (2025PHB253-001).

### 2.2 Participants

Consecutive patients who underwent simultaneous UWF-cSLO and SS-OCT imaging at our center between April and December 2025 were screened. Inclusion required age ≥18 years and adequate image quality in both modalities. Eyes were excluded if they had significant media opacity (cataract graded LOCS III ≥ N3, vitreous haemorrhage, or dense corneal scar), retinal detachment, full-thickness macular hole, or dense submacular scar. Both eyes of bilateral participants were included. Image quality was assessed at acquisition by the operating technician and verified by a reading grader. cSLO images were required to have adequate, even illumination across the 135° field, and no blink or motion artefact crossing the posterior pole; SS-OCT scans were required to have a signal strength of ≥8/10 with a visible choroid–scleral interface. Eyes failing either modality were excluded. On this basis, 151 eyes were excluded because of inadequate image quality in either modality.

### 2.3 Image acquisition

All imaging was performed in a darkened room without pharmacological mydriasis unless clinically indicated. An integrated cSLO–SS-OCT platform (SKY; MicroClear Medical, Suzhou, China) equipped with five laser channels (488, 520, 665, 785, and 1,060 nm) acquired UWF-cSLO and SS-OCT data simultaneously in a single session. A 135° fundus image was captured across four channels (488, 520, 665, and 785 nm); only the R-channel (665 nm) and IR-channel (785 nm) images were retained for analysis. SS-OCT data were obtained at 1,060 nm using a standardised eight-line radial protocol centered on the fovea, each line spanning 28 mm.

### 2.4 Image preprocessing and AI-based choroidal vessel density quantification

Preprocessing comprised region-of-interest (ROI) extraction, retaining the fundus area, followed by contrast-limited adaptive histogram equalisation (CLAHE; 8 × 8 tile grid, contrast limit 1.2) to enhance structural detail while minimising noise amplification. Choroidal vessels were segmented from UWF-cSLO images with a TransUNet encoder–decoder architecture trained on manually annotated images and evaluated on a held-out test set by the Dice similarity coefficient, pixel-level sensitivity, and specificity. Ground-truth annotations were generated on 144 original R-channel and 147 original IR-channel images: two fellowship-trained retinal specialists (JEZ and CY) with experienced retinal image grading, masked to clinical and OCT data, independently annotated choroidal vessels using ITK-SNAP (version 3.8), and discordant regions were adjudicated by a third senior retinal specialist (SYY), whose annotation served as the final ground truth. The inter-grader agreement between the two primary graders was high (mean Dice = 0.895). The annotated images were then partitioned into patches, yielding 1,296 R-channel and 1,323 IR-channel images for model training. The entire dataset was randomly partitioned into a training-test subset and a validation subset following an 8:2 ratio. The partition was performed at the image level because segmentation is a pixel-level task that incorporates no participant-level information; a participant-level split is planned for future multi-center external-validation study. Data augmentation strategies were employed in the model training process, and cross-validation was not used in this study. Specifically, the training set included 1,323 images for the IR channel and 1,296 images for the R channel. Each processed image yielded a binary segmentation map from which CVD was calculated:

CVD=SegmentedChoroidalVesselArea/TotalROIArea

R-CVD and IR-CVD were derived separately from the respective channel images. A combined metric, IR + R CVD, was obtained by summing R-CVD and IR-CVD.

The ROI encompassed the full fundus field after automated exclusion of the optic-disc margin. Workflows for image preprocessing and model development are illustrated in Figures 1, 2, respectively.

**Figure 1:**
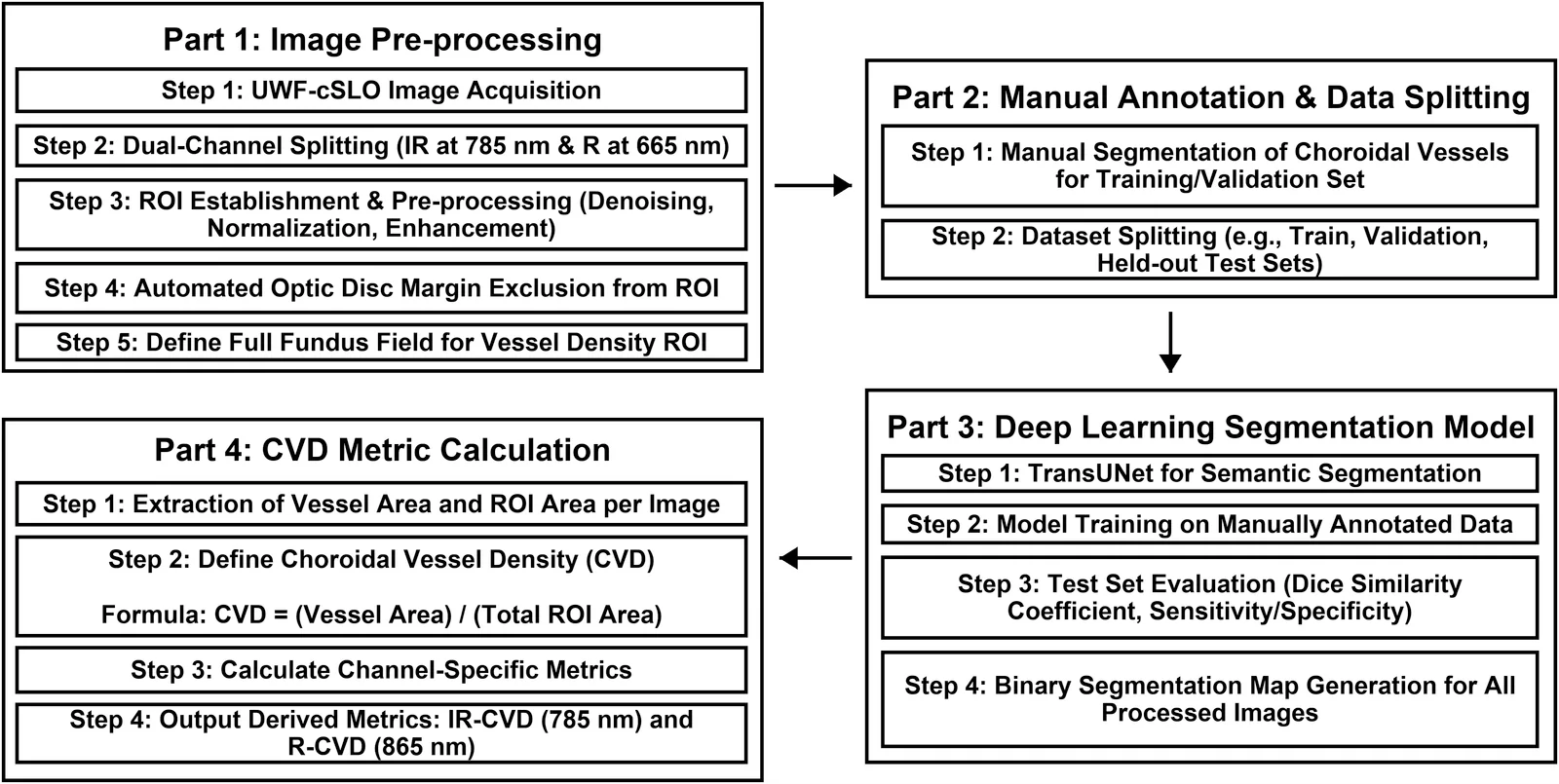
Workflow for image preprocessing and AI model training.

**Figure 2:**
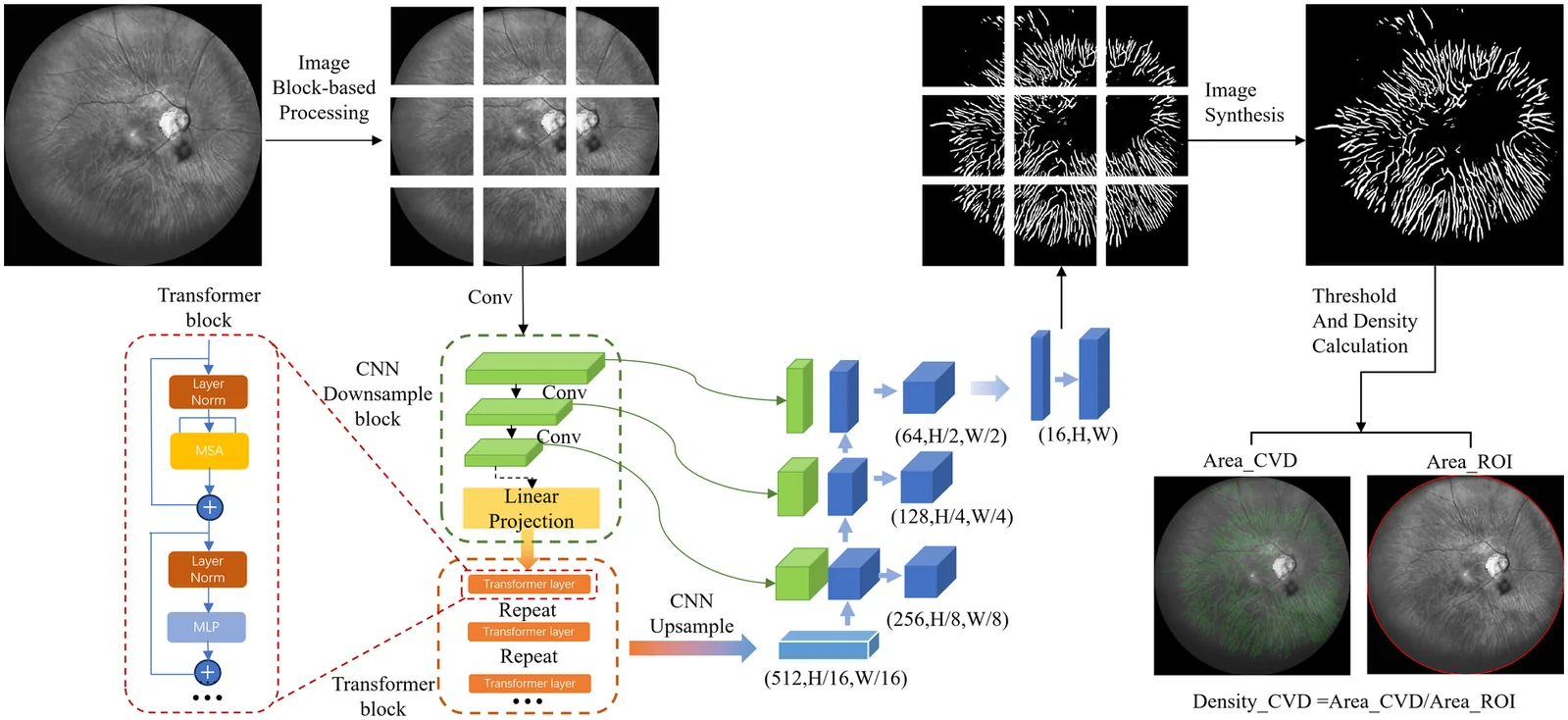
Workflow of TransUNet encoder–decoder architecture.

### 2.5 SS-OCT choroidal parameters

Eight parameters were extracted automatically from the radial SS-OCT scans: CVI (LA/TCA), TCA, LA, SA, CT, vessel volume index (VVI), choroidal vascular volume (CVV), and choroidal volume (CV). Notably, CT in this study denotes the mean choroidal thickness averaged across the entire 28 mm scan line rather than a single subfoveal point measurement, an approach adopted to capture the overall choroidal status of the eye more comprehensively. All eight radial scan lines contributed to quantification. Each parameter was computed per scan line and averaged across the eight lines, and no parameter was derived from the subfoveal line alone.

### 2.6 Statistical analysis

Continuous variables are reported as median (interquartile range (IQR) and categorical variables as count (percentage). Spearman rank correlation coefficients (*ρ*) were computed between each CVD metric and each OCT parameter. To account for inter-eye correlation in bilateral participants, 95% confidence intervals were estimated by participant-level clustered bootstrap resampling, and cluster-adjusted *p*-values were obtained from generalised estimating equations (GEE) with an exchangeable working correlation structure. Multivariable GEE models adjusted for age and sex were fitted for all 16 CVD–OCT pairs; CVD values were standardised to *z*-scores to permit direct effect-size comparison. All *p*-values were corrected by the Benjamini–Hochberg false-discovery-rate (FDR) procedure at *q* < 0.05. Analyses were conducted in Python 3 using SciPy and Statsmodels. As a sensitivity analysis for the ROC thresholds, classification was repeated using quartile-based thresholds (the highest vs. the lowest quartile, and each extreme quartile vs. the remainder of the cohort). To verify that bilateral inclusion did not inflate the associations, all CVD–OCT correlations were additionally recomputed across 1,000 one-eye-per-participant resamples.

## 3 Results

### 3.1 Baseline characteristics

A total of 633 eyes from 354 participants met the inclusion criteria (Table 1); 279 participants (78.8%) contributed bilateral data. The median age was 56.0 years (IQR 42.0–66.0). The cohort comprised 346 (54.7%) healthy eyes, 82 (13.0%) myopic eyes, 61 (9.6%) eyes with AMD, 5 (0.8%) eyes with pachychoroid spectrum disease, 74 (11.7%) eyes with diabetic retinopathy, and 65 (10.3%) eyes with other retinal diseases (e.g., epiretinal membrane).

**Table 1:** Baseline characteristics of participants.

Variable	Overall
Eyes	633
Participants	354
Bilateral contributors, *n* (%)	279 (78.8%)
Age, years	56.0 (42.0–66.0)
Age <40 years, *n* (%)	130 (20.54%)
Age 40–60 years, *n* (%)	266 (42.02%)
Age >60 years, *n* (%)	237 (37.44%)
Male sex, *n* (%)	267 (42.2%)

Median R-CVD was 0.009 (IQR 0.002–0.032), substantially lower than IR-CVD at 0.034 (IQR 0.017–0.058). Median CVI was 0.558 (IQR 0.540–0.579), and median CT was 223.3 μm (IQR 188.1–265.6). Descriptive statistics for all cSLO and SS-OCT metrics are provided in Table 2.

**Table 2:** Descriptive statistics of cSLO choroidal vessel metrics and OCT parameters.

SS-OCT parameter	Median	IQR
IR CVD	0.0337	0.0170–0.0578
R CVD	0.0094	0.0019–0.0318
IR + R CVD	0.0509	0.0261–0.0839
bscan_Mean_CVI	0.5578	0.5401–0.5792
bscan_Mean_TCA (mm^2^)	4.9138	4.1375–5.8438
bscan_Mean_LA (mm^2^)	2.76	2.2850–3.2850
bscan_Mean_SA (mm^2^)	2.1525	1.8313–2.5425
bscan_Mean_CT (μm)	223.3175	188.0625–265.5725
VVI	0.5502	0.5304–0.5692
CVV (mm^3^)	43.38	36.3600–49.7800
CV (mm^3^)	77.96	67.3100–90.3600

### 3.2 AI-assisted segmentation performance

The TransUNet model achieved consistently high segmentation performance in both channels, with all metrics exceeding 0.95 (Table 3). Representative qualitative examples spanning the performance spectrum—including an excellent segmentation (Dice = 0.98) and a relatively poor segmentation (Dice = 0.80)—are presented in Supplementary Figure S1.

**Table 3:** Performance of the AI model.

Channel	MPA	mIoU	mDice	mSen	mSpe
IR	0.9685	0.9519	0.9689	0.9701	0.9677
R	0.9675	0.9509	0.9675	0.9665	0.9670

MPA, mean pixel accuracy; mIoU, mean intersection over union; mDice, mean dice coefficient; mSen, mean sensitivity; mSpe, mean specificity.

### 3.3 Correlation analysis

Of the 40 CVD-OCT association tests (24 Spearman correlations and 16 multivariable GEE models), 28 (70%) remained significant after FDR correction (*q* < 0.05). The full correlation matrix is presented in Figure 3.

**Figure 3:**
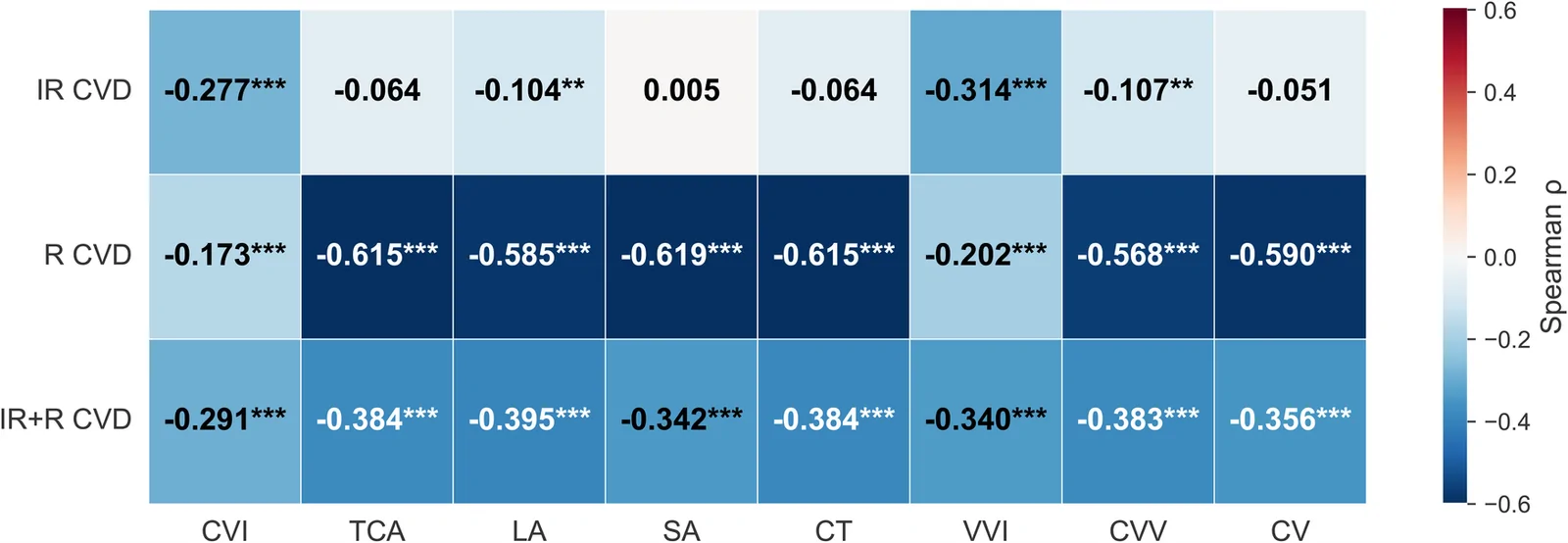
Heatmap of associations between cSLO choroidal vessel density and SS-OCT-derived choroidal parameters. ***p < 0.001, **p < 0.01, *p < 0.05.

R-CVD showed the strongest inverse associations with structural and volumetric OCT parameters (Table 4): SA (*ρ* = −0.619), CT (*ρ* = −0.615), and TCA (*ρ* = −0.615; all *q* < 0.001). Correlations with proportion-based indices were comparatively modest (CVI: *ρ* = −0.173; VVI: *ρ* = −0.202; *q* < 0.001), indicating that the R-channel signal reflects absolute choroidal dimensions more than the vascular-to-stromal ratio. The full set of R-CVD associations is presented in the Table 4.

**Table 4:** Associations between R CVD and SS-OCT choroidal parameters.

SS-OCT parameter	Spearman *ρ* (95% CI)	*p* value
CT, μm	−0.615 (−0.674 to −0.543)	<0.001
LA, mm^2^	−0.585 (−0.642 to −0.510)	<0.001
SA, mm^2^	−0.619 (−0.677 to −0.551)	<0.001
TCA, mm^2^	−0.615 (−0.674 to −0.543)	<0.001
CVV, mm^3^	−0.568 (−0.637 to −0.493)	<0.001
CV, mm^3^	−0.590 (−0.657 to −0.508)	<0.001
CVI	−0.173 (−0.287 to −0.095)	<0.001
VVI	−0.202 (−0.278 to −0.123)	<0.001

Correlation coefficients were estimated using Spearman rank correlation. Confidence intervals were derived using participant-level clustered bootstrap resampling.

The combined IR + R CVD correlated negatively with all OCT parameters (*ρ* range −0.29 to −0.40) and yielded relatively stronger associations with proportion-based indices than R-CVD alone, notably CVI (*ρ* = −0.29) and VVI (*ρ* = −0.34), suggesting that dual-channel integration captures complementary vascular information.

IR-CVD in isolation exhibited the weakest effect sizes. Significant but modest correlations emerged for CVI (*ρ* = −0.28) and VVI (*ρ* = −0.31), whereas most structural parameters did not reach significance, constraining its standalone predictive value.

The one-eye-per-participant sensitivity analysis yielded correlation coefficients essentially identical to the full-data estimates (Supplementary Table S1), indicating that bilateral inclusion did not materially influence the associations.

### 3.4 Multivariable adjusted analysis

Because unadjusted correlations may be confounded by demographic factors, all CVD–OCT associations were re-examined in GEE models adjusted for age and sex. Effect sizes were attenuated but remained significant, and the channel-dependent pattern persisted (Table 5).

**Table 5:** Multivariable adjusted GEE analysis strongest adjusted cSLO metric (adjusted for age and sex).

cSLO metric	OCT parameter	Adjusted *β* (95% CI)	*p*-value
R CVD	SA	−0.388 (−0.472 to −0.304)	<0.001
R CVD	TCA	−0.363 (−0.444 to −0.281)	<0.001
R CVD	CT	−0.363 (−0.444 to −0.281)	<0.001
R CVD	LA	−0.342 (−0.420 to −0.264)	<0.001
R CVD	CV	−0.365 (−0.444 to −0.286)	<0.001
R CVD	CVV	−0.352 (−0.429 to −0.275)	<0.001
R CVD	CVI	−0.230 (−0.344 to −0.115)	<0.001
R CVD	VVI	−0.257 (−0.375 to −0.139)	<0.001
IR + R CVD	VVI	−0.347 (−0.462 to −0.231)	<0.001
IR + R CVD	CVI	−0.313 (−0.423 to −0.204)	<0.001
IR + R CVD	CVV	−0.315 (−0.384 to −0.247)	<0.001

R-CVD remained the strongest predictor of structural and volumetric parameters (SA, TCA, CT, LA, CV, and CVV), whereas IR + R CVD was most closely associated with proportion-based indices (CVI and VVI). The persistence of channel-dependent patterns after covariate adjustment supports the view that these associations are driven by intrinsic optical–anatomical relationships rather than demographic confounding alone.

### 3.5 Receiver operating characteristic analysis

ROC analysis assessed the capacity of each CVD metric to classify eyes above vs. below the median for each OCT parameter (Figure 4; Table 6). R-CVD discriminated thick from thin choroids with an AUC of 0.789 (95% CI 0.753–0.828), sensitivity of 72.5%, and specificity of 74.8%. IR-CVD, by contrast, showed near-chance performance (AUC 0.517; 95% CI 0.469–0.562). Quartile-based sensitivity analyses confirmed that discrimination was not an artefact of the median threshold: for R-CVD, AUCs ranged from 0.76 to 0.81 when each extreme quartile was contrasted with the remainder of the cohort, and from 0.88 to 0.92 when the highest quartile was contrasted with the lowest (CT: AUC 0.908, 95% CI 0.866–0.947; Supplementary Table S2 and Supplementary Figure S2).

**Figure 4:**
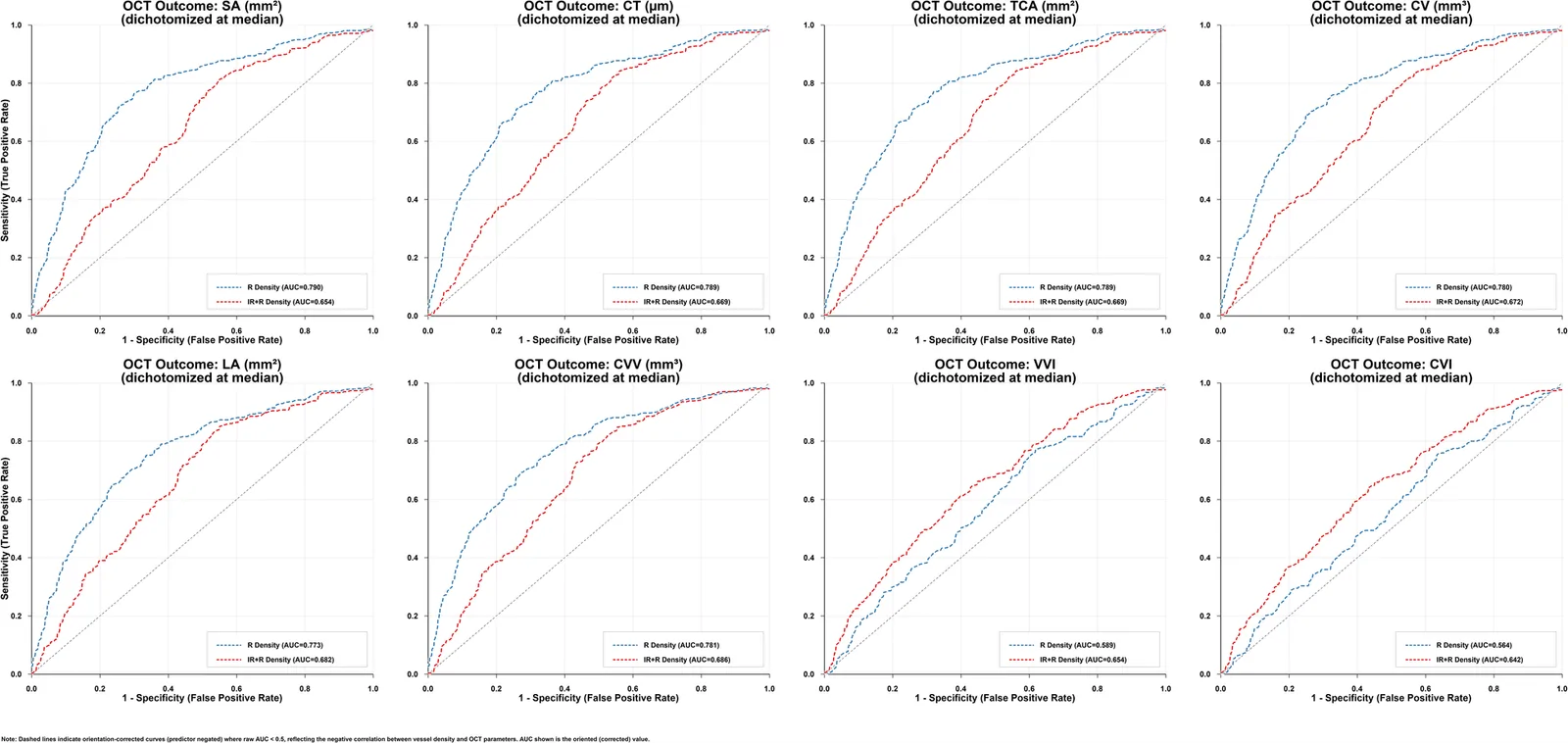
ROC analysis of cSLO choroidal vessel density to predict above-median OCT parameters.

**Table 6:** ROC analysis: choroidal vessel density for predicting above-median OCT parameters.

OCT parameter	cSLO	Cutoff	AUC	Sensitivity	Specificity
SA (mm^2^)	R CVD	2.15	0.790	0.790	0.698
CT (μm)	R CVD	223.32	0.789	0.725	0.748
TCA (mm^2^)	R CVD	4.91	0.789	0.725	0.748
CVV (mm^3^)	R CVD	43.38	0.781	0.707	0.740
CV (mm^3^)	R CVD	77.96	0.780	0.717	0.748
LA (mm^2^)	R CVD	2.76	0.773	0.702	0.734
CVV (mm^3^)	IR + R CVD	43.38	0.686	0.844	0.476
LA (mm^2^)	IR + R CVD	2.76	0.682	0.847	0.476
CV (mm^3^)	IR + R CVD	77.96	0.672	0.831	0.463
CT (μm)	IR + R CVD	223.32	0.669	0.838	0.466
TCA (mm^2^)	IR + R CVD	4.91	0.669	0.838	0.466
SA (mm^2^)	IR + R CVD	2.15	0.654	0.829	0.458
VVI	IR + R CVD	0.55	0.654	0.662	0.569
VVI	IR CVD	0.55	0.643	0.701	0.532
CVI	IR + R CVD	0.56	0.642	0.659	0.565
CVI	IR CVD	0.56	0.636	0.702	0.518

## 4 Discussion

In this study, AI-based choroidal vessel segmentation from UWF-cSLO images achieved uniformly high accuracy (all metrics > 0.95) in both the IR and R channels, and the resulting CVD values correlated strongly and consistently with eight SS-OCT choroidal parameters. To our knowledge, this represents the first derivation and cross-modal validation of a quantitative, AI-driven CVD metric from UWF-cSLO.

Over the past decade, choroidal assessment from fundus images has progressed from subjective tessellation grading to semi-automated quantification (6, 7, 13, 14). Early studies relied on subjective grading by clinicians, which was inherently grader-dependent and poorly reproducible (14). Yoshihara et al. proposed tessellated fundus indices (TFIs) derived from RGB-pixel ratios on CFP (7), and Shao et al. (6) introduced FTD through AI-based segmentation of exposed choroidal area. Both approaches, however, depend on broadband white-light CFP, which is susceptible to transillumination artefacts and cannot selectively image deep choroidal structures. By contrast, cSLO employs monochromatic laser excitation at specific wavelengths that penetrate tissue more deeply and suffer less attenuation from RPE melanin absorption than white-light sources. The confocal pinhole further suppresses out-of-focus scattered light, sharpening the contrast of choroidal vasculature. These optical properties enable cSLO-derived CVD to delineate vascular features that are either obscured or confounded by overlying retinal layers on CFP-based tessellation metrics, constituting a meaningful advance in non-OCT choroidal imaging.

Concurrently, OCT-based CVI has been established as a reliable choroidal biomarker. Agrawal et al. (3) reported a normative subfoveal CVI of approximately 65.6% in healthy eyes, and subsequent work has confirmed that CVI varies less than CT across physiological states (15). CVI measurement nonetheless requires dedicated OCT hardware, offline binarisation with software such as ImageJ, and lacks cross-study standardisation (2, 16). The present study addresses these constraints by deriving CVD directly from UWF-cSLO images with a TransUNet model and validating it against eight SS-OCT parameters. Because the cSLO and SS-OCT data were acquired simultaneously on a single integrated platform by a same scan, spatial co-registration was inherently precise, strengthening the reliability of the cross-modal comparisons.

The median CVI in our cohort (0.558) falls below the 0.656 reported by Agrawal et al. in healthy Indian adults. Several factors likely account for this difference. Our population was predominantly East Asian, with a higher median age (56 years) and potentially distinct choroidal melanin content and stromal composition. In addition, inter-study variation in the binarisation algorithm, OCT device, and scan protocol is well recognised as a source of CVI heterogeneity. These methodological differences warrant consideration when comparing CVI values across studies.

A note on CVD nomenclature is appropriate here. CVD, as defined in this study, quantifies the area of choroidal vessels visible on cSLO relative to the total fundus ROI; it is therefore an index of vessel visibility rather than of absolute vascular abundance. Visibility is enhanced when the overlying tissue is thin or attenuated—conditions that correspond to reduced CT, SA, and TCA on OCT—because less tissue separates the choroidal vessels from the imaging plane. Conversely, in eyes with thick choroids and dense stroma, vessels lie in a deeper, more optically opaque tissue bed and produce lower cSLO contrast despite being volumetrically larger. CVD should accordingly be interpreted as a composite optical metric that integrates the effects of tissue depth, RPE integrity, and stromal density on vessel contrast, rather than as a direct histological measure of vascular density.

Two distinct mechanisms are needed to explain the full correlation pattern. The inverse association between CVD and OCT parameters follows directly from the visibility principle. Greater vessel contrast—and thus higher CVD—arises preferentially when the choroid is thin, the RPE attenuated, or the stroma sparse, each corresponding to lower CT, LA, SA, and TCA on SS-OCT. The physical basis is well established: near-infrared wavelengths penetrate the sensory retina readily because melanin, water, macular pigment, and haemoglobin absorb less at longer wavelengths (12), while the confocal aperture rejects multiply scattered photons to sharpen deep-layer contrast (10, 12).

This visibility framework directly explains the inverse associations with absolute dimensions, but not those with the proportion-based indices CVI and VVI. The latter are better understood as reflecting concomitant vascular remodelling. Progressive choroidal thinning is accompanied by disproportionate luminal loss—luminal area contracts proportionally more than stromal area, thus CVI and VVI decline with thinning. The eyes with the most visible vessels (high CVD) are frequently the same eyes with luminal-predominant rarefaction (low CVI and VVI). Consistent with this indirect pathway, the CVD–CVI/VVI associations were markedly weaker (|*ρ*| ≈ 0.17–0.20) than the CVD–structural associations, and CVD should not be interpreted as a direct surrogate for CVI.

The two channels displayed divergent correlation profiles. The R channel (665 nm), with its shorter wavelength and shallower penetration, delineated high-contrast vessel edges in thinned choroids and correlated most strongly with absolute structural metrics (CT *ρ* = −0.615; SA *ρ* = −0.619; TCA *ρ* = −0.615). The IR channel (785 nm), penetrating deeper, interacted more broadly with the choroidal vascular bed more completely and correlated preferentially with proportional indices (CVI *ρ* = −0.28, VVI *ρ* = −0.31) rather than R-CVD for those indices (CVI *ρ* = −0.17; VVI *ρ* = −0.20). This is the direction expected from multi-wavelength imaging theory, in which longer wavelengths visualise progressively deeper stromal/luminal structure. Combining both channels strengthened the association with CVI (*ρ* = −0.29) and VVI (*ρ* = −0.34), consistent with the multi-wavelength imaging principle whereby longer wavelengths visualise progressively deeper structures (17).

The wavelength-dependent optical basis of these divergent correlation profiles is illustrated schematically in Figure 5. Furthermore, the consecutive sampling strategy and inclusion of cases with multiple diagnoses indicate that the observed correlations reflect a broad clinic-based spectrum of choroidal features rather than a single disease entity. Disease-specific subgroup analyses are needed in future studies to further clarify these associations.

**Figure 5:**
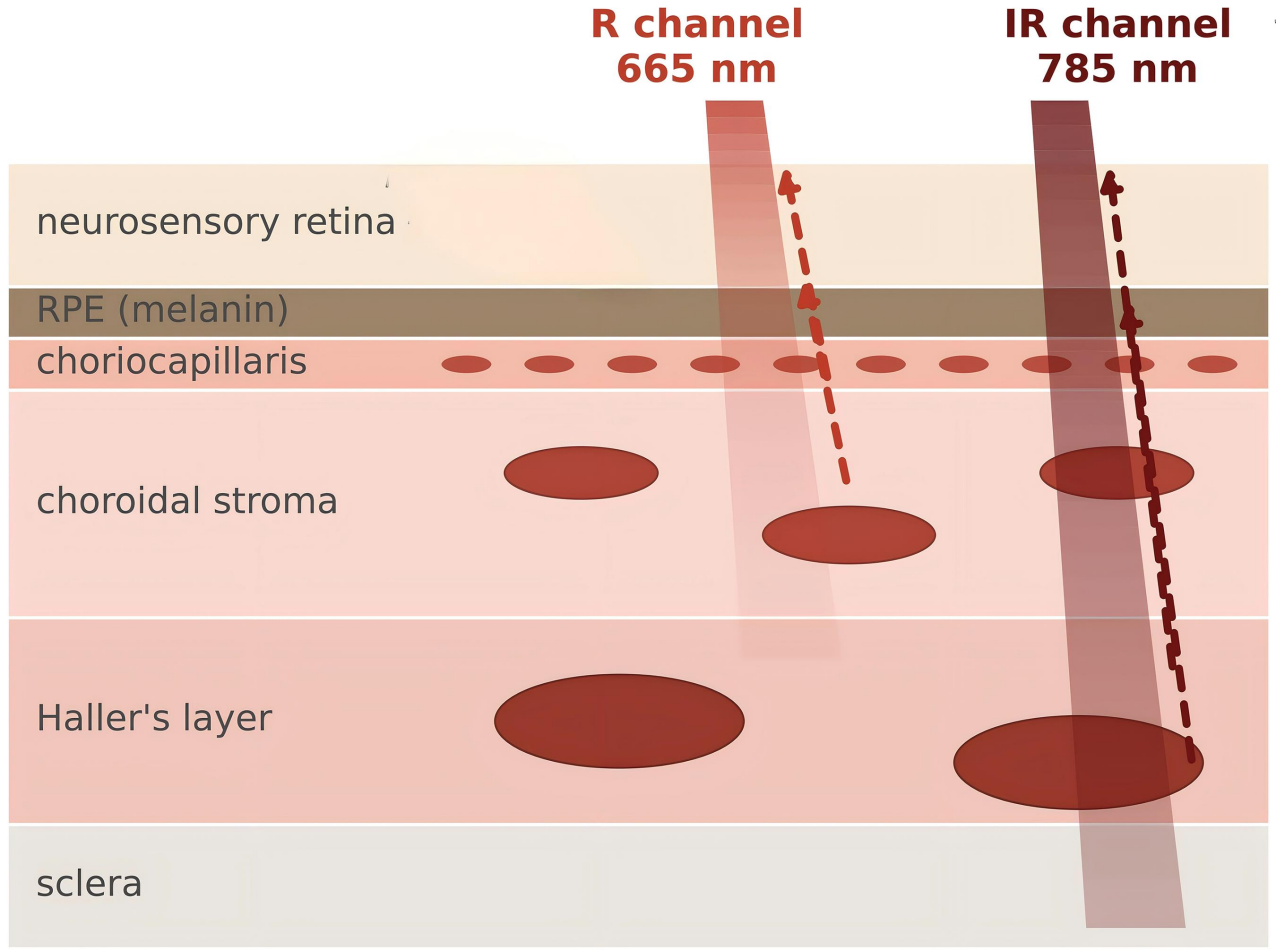
Wavelength-dependent optical sampling explains the divergent correlation profiles of R-CVD and IR-CVD.

These observations align with tessellation research demonstrating that progressive fundus tessellation reflects genuine choroidal vascular loss: as tessellation grade rises, CVI, LA, and TCA decline, with luminal area contracting proportionally more than stroma (8). Our findings extend this body of evidence by showing that cSLO-derived CVD captures the same structural changes without recourse to OCT.

CT in the present study was defined as the mean thickness across the full 28 mm scan line rather than the subfoveal point value reported in most prior work. Because choroidal thickness tapers progressively from the fovea toward the periphery, scan-line averaging yields lower absolute values. No normative reference range has been established for this metric, and direct numerical comparison with published subfoveal CT data should be undertaken with caution.

From a translational standpoint, cSLO platforms are substantially less expensive than SS-OCT systems and are already deployed in many primary-care and screening settings. Extracting CVD from routine cSLO images could therefore enable choroidal risk stratification without dedicated OCT hardware or additional acquisition protocols. The discriminative performance of R-CVD for thin vs. thick choroids (AUC 0.789; sensitivity 72.5%; specificity 74.8%) supports its feasibility as a screening metric in resource-constrained environments. Furthermore, large retrospective cSLO archives could be reprocessed with the present AI pipeline to facilitate population-level choroidal phenotyping where prospective OCT data are unavailable. AI-derived CVD could also be appended to existing fundus-based deep-learning pipelines as a channel-derived feature, potentially enriching risk prediction for conditions such as pathological myopia without hardware modification.

This study has several limitations. First, all images were acquired using a single device at one center. Therefore, the generalizability of the segmentation model and CVD metric across different cSLO platforms, acquisition protocols, and populations remains uncertain. External validation in multi-center, multi-device cohorts is required before clinical application.

Second, the segmentation model was split at the image level rather than the participant level. Although segmentation is a pixel-level task without direct use of participant information, fellow eyes are not fully independent. Participant-level splitting is therefore a more conservative strategy for future studies.

Third, only age and sex were available as covariates. Important determinants of choroidal thickness, including refractive error, diurnal variation, blood pressure, and systemic vascular status, were unavailable in this retrospective dataset. Axial elongation may thin the choroid, increase tessellation, and partially contribute to the inverse association between CVD and CT. Diurnal variation is unlikely to fully explain the cross-modal correlations because cSLO and SS-OCT were obtained during the same session, although acquisition time was not standardized. Unmeasured systemic factors may still introduce residual confounding. Future prospective studies should incorporate biometric measurements and systemic phenotyping.

Fourth, ROC analyses dichotomized OCT parameters using cohort medians. Although median splitting is data-driven and may reduce information compared with clinically defined thresholds, no validated cut-offs currently exist for scan-line-averaged choroidal parameters. Therefore, median-based analyses were used as the primary approach, with robustness confirmed by quartile-based sensitivity analyses (Supplementary Table S2, Supplementary Figure S2). Future studies should evaluate R-CVD using clinically established thresholds.

Finally, the cross-sectional design prevents causal inference. In addition, cSLO infrared reflectance may be influenced by choroidal thickness, as shown in geographic atrophy (18). Therefore, cSLO-derived CVD should be interpreted together with OCT parameters. Longitudinal studies are needed to determine whether CVD can reflect choroidal remodelling over time.

## 5 Conclusion

AI-based choroidal vessel segmentation from UWF-cSLO yields a reliable and clinically accessible CVD metric that correlates strongly with SS-OCT choroidal parameters. Channel-dependent correlation patterns illuminate the wavelength-specific interaction between laser light and choroidal tissue. Pending validation in prospective, multi-center cohorts, this approach has the potential to extend quantitative choroidal assessment from specialised OCT facilities to routine fundus-imaging settings.

## Data Availability

The raw data supporting the conclusions of this article will be made available by the authors, without undue reservation.

## Supplementary material

Supplementary Figure S1 Representative qualitative segmentation examples spanning the performance spectrum. **(A)** Represents excellent segmentation (Dice = 0.98) and **(B)** represents a relatively poor segmentation (Dice = 0.80). with white arrows indicating the choroidal vascular regions that failed to be automatically segmented.

Supplementary Figure S2 ROC curves for R-CVD discriminating choroidal thickness under median and quartile-based thresholds.

Supplementary Table S1 One-eye-per-participant sensitivity analysis of CVD-OCT Spearman correlations (1,000 resampling iterations; n = 354 eyes per iteration).

Supplementary Table S2 Quartile-threshold ROC sensitivity analysis for R-CVD (AUCs with participant-level clustered bootstrap 95% CIs).
