# Supplementary material for: Beyond tessellation: AI-derived choroidal vessel density on multi-channel cSLO as a surrogate biomarker for OCT choroidal parameters
Source: Front Med (Lausanne). 2026 Oct 7;13:1884151. doi: 10.3389/fmed.2026.1884151 (PMC13683970; doi:10.3389/fmed.2026.1884151)
Supplement: Supplementary file 1 [file Table1.docx]

**Supplementary Table S1.** One-eye-per-participant sensitivity analysis of CVD–OCT Spearman correlations (1,000 resampling iterations; n = 354 eyes per iteration).

| **cSLO metric** | **OCT parameter** | **Full-data Spearman ρ** | **One-eye-per-participant median ρ (95% range)** |
| --- | --- | --- | --- |
| R-CVD | CT | −0.597 | −0.605 (−0.638 to −0.573) |
| R-CVD | SA | −0.597 | −0.605 (−0.638 to −0.574) |
| R-CVD | TCA | −0.597 | −0.605 (−0.638 to −0.573) |
| R-CVD | LA | −0.574 | −0.583 (−0.618 to −0.551) |
| R-CVD | CVV | −0.559 | −0.567 (−0.603 to −0.534) |
| R-CVD | CV | −0.580 | −0.588 (−0.621 to −0.556) |
| R-CVD | CVI | −0.193 | −0.207 (−0.256 to −0.161) |
| R-CVD | VVI | −0.197 | −0.214 (−0.259 to −0.169) |
| IR-CVD | CVI | −0.277 | −0.280 (−0.323 to −0.232) |
| IR-CVD | VVI | −0.314 | −0.309 (−0.351 to −0.266) |
| IR+R CVD | CVI | −0.291 | −0.301 (−0.347 to −0.260) |
| IR+R CVD | VVI | −0.339 | −0.343 (−0.388 to −0.299) |
