# Supplementary material for: Beyond tessellation: AI-derived choroidal vessel density on multi-channel cSLO as a surrogate biomarker for OCT choroidal parameters
Source: Front Med (Lausanne). 2026 Oct 7;13:1884151. doi: 10.3389/fmed.2026.1884151 (PMC13683970; doi:10.3389/fmed.2026.1884151)
Supplement: Supplementary file 2 [file Table2.docx]

**Supplementary Table S2.** Quartile-threshold ROC sensitivity analysis for R-CVD (AUCs with participant-level clustered bootstrap 95% CIs).

| **OCT parameter** | **Comparison** | **Quartile cut-offs (Q3/Q1)** | **AUC (95% CI)** |
| --- | --- | --- | --- |
| CT (μm) | ≥Q3 vs ≤Q1 (extreme groups) | 265.6 / 188.1 | 0.908 (0.866–0.947) |
| CT (μm) | ≤Q1 vs remainder |  | 0.801 (0.749–0.847) |
| CT (μm) | ≥Q3 vs remainder |  | 0.805 (0.767–0.846) |
| SA (mm²) | ≥Q3 vs ≤Q1 (extreme groups) | 2.54 / 1.83 | 0.916 (0.878–0.952) |
| SA (mm²) | ≤Q1 vs remainder |  | 0.813 (0.763–0.858) |
| SA (mm²) | ≥Q3 vs remainder |  | 0.797 (0.759–0.836) |
| TCA (mm²) | ≥Q3 vs ≤Q1 (extreme groups) | 5.84 / 4.14 | 0.908 (0.866–0.947) |
| TCA (mm²) | ≤Q1 vs remainder |  | 0.801 (0.749–0.847) |
| TCA (mm²) | ≥Q3 vs remainder |  | 0.805 (0.767–0.846) |
| LA (mm²) | ≥Q3 vs ≤Q1 (extreme groups) | 3.29 / 2.29 | 0.892 (0.847–0.932) |
| LA (mm²) | ≤Q1 vs remainder |  | 0.783 (0.730–0.829) |
| LA (mm²) | ≥Q3 vs remainder |  | 0.800 (0.761–0.841) |
| CVV (mm³) | ≥Q3 vs ≤Q1 (extreme groups) | 49.78 / 36.36 | 0.883 (0.837–0.926) |
| CVV (mm³) | ≤Q1 vs remainder |  | 0.762 (0.711–0.813) |
| CVV (mm³) | ≥Q3 vs remainder |  | 0.795 (0.755–0.837) |
| CV (mm³) | ≥Q3 vs ≤Q1 (extreme groups) | 90.36 / 67.31 | 0.891 (0.846–0.933) |
| CV (mm³) | ≤Q1 vs remainder |  | 0.771 (0.719–0.822) |
| CV (mm³) | ≥Q3 vs remainder |  | 0.801 (0.762–0.841) |
